# IMPORTANCE trial: a provisional study-design of a single-center, phase II, double-blinded, placebo-controlled, randomized, 4-week study to compare the efficacy and safety of intranasal esketamine in chronic opioid refractory pain

**DOI:** 10.12688/f1000research.27809.1

**Published:** 2021-01-22

**Authors:** Mauricio Fernandes, Magdalena Schelotto, Philipp Maximilian Doldi, Giovanna Milani, Abul Andrés Ariza Manzano, Doriam Perera Valdivia, Alexandra Marie Winter Matos, Yasmin Hamdy Abdelrahim, Shaza Ahmed Hamad Bek, Benito K. Benitez, Vanessa Luiza Romanelli Tavares, Abdulrahim M. Basendwah, Luis Henrique Albuquerque Sousa, Naiara Faria Xavier, Tania Zertuche Maldonado, Sarah Toyomi de Oliveira, Melisa Chaker, Michelle Menon Miyake, Elif Uygur Kucukseymen, Kinza Waqar, Ola M.J. Alkhozondar, Ricardo Bernardo da Silva, Guilhermo Droppelmann, Antonio Vaz de Macedo, Rui Nakamura, Felipe Fregni

**Affiliations:** 1 Dresden International University, Dresden, Germany; 2 Universidad de la República (UdelaR)., Montevideo, Uruguay; 3 Universitätsklinikum München Campus Großhadern, München, Germany; 4 ABC School of Medicine, Sao Paulo, Brazil; 5 Colegio Mayor de Nuestra Señora del Rosario, Bogota, Colombia; 6 National Autonomous University of Nicaragua, Manangua, Nicaragua; 7 Universidad Iberoamericana (UNIBE), Santo Domingo, Dominican Republic; 8 Department of Old Age Psychiatry, Doha, Qatar; 9 National Center for Cancer Care and Research, Doha, Qatar; 10 Department of Oral and Craniomaxillofacial Surgery, Basel, Switzerland; 11 Human Genome and Stem Cell Research Center, Sao Paulo, Brazil; 12 Oncology Division, Internal Medicine Department, King Fahad Armed Forces Hospital, Jeddah, Saudi Arabia; 13 Neurosurgery Department, University of Sao Paulo (USP), Sao Paulo, Brazil; 14 Ophthalmology Department, University of Sao Paulo, Sao Paulo, Brazil; 15 Tecnológico de Monterrey, Escuela de Medicina Y Ciências de la Salud, Monterrey, Mexico; 16 University of Sao Paulo (USP), Sao Paulo, Brazil; 17 Department of Pediatric Cardiology, Hospital Nacional de Pediatría Juan Pedro Garrahan, Buenos Aires, Argentina; 18 Department of Otolaryngology, Massachusetts Eye and Ear Infirmary, Boston, USA; 19 Neuromodulation Center and Center for Clinical Research Learning. Spaulding Rehabilitation Hospital, Massachusetts General Hospital, Boston, USA; 20 National University of Sciences and Technology, Islamabad, Pakistan; 21 Department of Pharmacy Clinical Informatics, Hamad Medical Corporation, Doha, Qatar; 22 Department of Vascular and Endovascular Surgery, Pontifical Catholic University of Paraná, Londrina, Brazil; 23 23- Academic Unit. Clinica MEDS., Santiago, Chile; 24 PPCR Program, ECPE, Harvard T.H. Chan School of Public Health, Boston, USA

**Keywords:** Cancer pain, Esketamine, Pain measurement, Opioid refractory

## Abstract

**Background:**  Cancer is the second leading cause of death globally. Up to 86% of advanced cancer patients experience significant pain, while 10-20% live in chronic pain. Besides, increasing prescription of opioids resulted in 33,000 deaths in the US in 2015. Both reduce patients’ functional status and quality of life. While cancer survival rates are increasing, therapeutic options for chronic opioid refractory pain are still limited. Esketamine is the s-enantiomer of ketamine, with superior analgesic effect and less psychotomimetic side effects. Intranasal esketamine was approved by the FDA for treatment-resistant depression. However, its use in chronic cancer pain has never been tested. Therefore, we propose a phase II, randomized, placebo-controlled trial to evaluate the efficacy and safety of intranasal esketamine in chronic opioid refractory cancer pain.

**Methods and analysis:** We will recruit 120 subjects with chronic opioid refractory pain, defined as pain lasting more than 3 months despite optimal therapy with high dose opioids (>60 mg morphine equivalent dose/day) and optimal adjuvant therapy. Subjects will be randomized into two groups: intranasal esketamine (56mg) and placebo. Treatment will be administered twice a week for four consecutive weeks. The primary outcome is defined as reduction in the Numeric Pain Rating Scale (NPRS) after first application. Secondary outcomes include NPRS reduction after four weeks, the number of daily morphine rescue doses, functional status and satisfaction, and depression.

**Conclusion:** This study may extend therapeutic options in patients with chronic pain, thus improving their quality of life and reducing opioid use.

**Trial registration:** Clinical Trials.gov, NCT04666623. Registered on 14 December 2020

## Introduction

Refractory pain is a challenging condition in Oncology. More than half of the patients with advanced stage cancer experienced pain ^1– 3^ and about 10 to 20% of the pain in cancer is refractory ^4^. The etiology is complex, with a mix of nociceptive, neuropathic and inflammatory mechanisms ^5^, resulting in a situation that can be difficult to control. Tolle *et al.*, described in 2000 that around 46% of family members of terminal patients referred that there is lack of adequate pain treatment when death is imminent ^6^. When opioids are not enough to relieve pain, it is necessary to associate an adjuvant, such as ketamine, as recommended by current National Comprehensive Cancer Network guidelines in pain not responding to other analgesics ^5, 7, 8^.

Ketamine was discovered in 1980, described as a non-competitive antagonist of N-methyl-D-aspartate (NMDA) receptor causing the inhibition of excitatory glutamate receptors in the central nervous system ^5^. It is commonly used as a general anesthetic agent, administered by intravenous or intramuscular route, but other usage and routes for this drug are being studied. Ketamine can cause not only dissociative anesthesia but also an analgesic and antidepressant effect ^5^. Low doses of ketamine, have minimal effect on cardiovascular and respiratory function, produce analgesia, modulate central sensitization, hyperalgesia and opioid tolerance ^9^.

One of the ketamine enantiomers largely studied nowadays for other potential use is esketamine, the most effective optical isomer of ketamine ^3^. The advantage of this enantiomer is that it induces less dissociative effects and more powerful analgesia and anesthesia ^10^. As an antidepressant, esketamine was approved by the FDA ^10^, and as an analgesic it is not labeled yet due to the lack of studies assessing its efficacy and safety in relieving pain ^10, 11^.

Intranasal esketamine use is approved by the FDA for refractory major depression ^12^. There are a couple of recently completed or ongoing trials testing intranasal esketamine for pain ^13, 14^. A pilot study recently published found similar results in analgesia between esketamine plus midazolam via intranasal, and intravenous morphine by patient controlled analgesia in spinal surgery patients ^13^.

Esketamine has shown promising results superior to those observed for ketamine due to its more potent analgesic effect, associated with less psychomimetic side effects referred above ^15, 16^. However, a large randomized controlled trial is needed to prove that this drug is effective to relieve pain, is safe, and causes a reduction in opioid consumption. 

Finding a better approach to manage severe and refractory cancer pain through rapid and efficient analgesia would redound to the benefits of the health care of these patients. Having an alternative to escalating doses of opioid would allow a reduction in opioid consumption in this population. This would decrease the risk of overdose and the frequent and unwanted side effects associated with high doses of these drugs. Opioid consumption is an epidemic health care concern in the United States and many other countries and efforts are strongly directed to combat it ^17^.

### Objectives

This research aims to assess the efficacy and safety of a four-week treatment with intranasal esketamine (56 mg) twice a week, combined with opioid analgesic and adjuvant standard therapy in the management of adult patients with severe and opioid refractory chronic cancer pain. ****

The primary aim is to investigate the analgesic efficacy of intranasal esketamine as an add-on therapy to potentially improve opioid resistant pain in cancer patients. We will evaluate whether intranasal esketamine alleviates chronic pain in this population by assessing the reduction with the Numeric Pain Rating Scale (NPRS) after the first dose of esketamine compared to baseline ^18^.

Our secondary aims are:

1. Assessment of safety ^19^ of this novel treatment. This will be measured by a self-reported and questionnaire-guided adverse events report.
2. Evaluation if esketamine treatment reduces the number of morphine rescues required daily by the patient.
3. To compare the patients’ functional status between both treatment groups.
4. To evaluate the response of depression score to esketamine therapy
5. To analyze if there is a difference in the efficacy of esketamine depending on the type of cancer and depression disorder of the patient.
6. To analyze the sustainability of the effect after each dose throughout the time of treatment.

### Trial design

We propose a single-center, randomized, placebo-controlled, phase II, double-blinded with two parallel groups, superiority trial. The primary endpoint will be the change in pain intensity measured by the NPRS ^18^ (at week 0, 1, 2, 3, and 4, measured exactly in between doses).

## Protocol

This is version 2 of the protocol from the 22 ^nd^ of December 2020.

### Study setting

The study will be conducted at a reference tertiary cancer hospital that will be selected based on willingness to participate and availability of qualified participants willing to take part in the study.

### Randomization

We plan to conduct a single hospital trial and randomization will be done by the OxMaR software. Allocation sequence will be concealed for everyone involved in the trial, except for the pharmacist and the nurse responsible for patient’s allocation. This allocation will remain hidden for health care professionals and participants until the final result of the study are obtained ^20^.

First approach will be performed by patient's physician and subsequent recruitment steps will be conducted by trained clinical staff. Written informed consent will be explained and signed. The recruiting physician will have access to a computer with OxMar software, the initial forms will be fulfilled and imputed personal data should be confirmed. The software will generate the allocation, which is automatically sent to the pharmacist that will provide the medication or placebo to the participant. The access to this allocation will be protected by Swordfish ( http://www.swordfishsecurity.com/).

The pharmacist will be responsible for formulating and packaging (identically) the trial medicines (placebo and esketamine nasal spray). He or she will label and deliver them with corresponding randomized number received from OxMar records.

### Blinding

All investigators, patients, care providers, outcome assessors, and study statisticians will remain blinded with respect to the treatment allocation throughout the trial. The masking will be assured by delivering identical unlabeled nasal injectors with the same volume (200 μL) and color of active drug and placebo. Each device will deliver 28mg of esketamine or placebo in a 200 μL solution, so in order to achieve the 56mg dose two devices will be required ^21, 22^.

Esketamine nasal spray has a certain taste often described as bitter, that can cause dysgeusia. Therefore, blinding of subjects will be further guaranteed by adding denatonium benzoate to the water-based placebo agent mimicking the taste of esketamine intranasal solution.

On each dosing day during the trial, participants will self-administer at 2 time points 1 spray of the study drug (esketamine or placebo) into each nostril. Each administration will be separated by 5 minutes. The administration will take place at the facility to ensure adherence and surveillance in regard to side effects.

***Emergency unblinding.*** In case of problems and safety concerns that cannot be solved with on-going blinded treatment, the participant’s allocated intervention will be revealed. Unblinding procedure will be performed at the request of the Data Monitoring Committee (DMC; see information below).

During the period of treatment, the patient's status will be evaluated weekly by an experienced blinded anesthesiologist. Vital signs and oxygen saturation levels will be examined and recorded. Also a physical examination will take place to follow up the patients’ functional status.

Sealed envelopes will be stored safely and access will be available 24/7 if needed. Treating physicians and the responsible pharmaceutical staff will be instructed and encouraged to maintain blinding status if possible. Certainly, they are advised to maintain blinding towards other subjects and ensure disclosure towards corporate sponsors, office staff, data analysts and study personnel.

The Investigator must report all code breaks (with reason) as they occur on the corresponding electronic case report form page. Unblinding should not necessarily be a reason for study drug discontinuation.

### Eligibility criteria

*Inclusion criteria*:

- Age ≥ 18 years; Male or female patients.
- Patients with refractory cancer pain, defined as: multiple evidence-based biomedical therapies used in a clinically appropriate and acceptable fashion have failed to reach treatment goals that mainly include adequate pain reduction and/or improvement in daily living functioning activities; AND/OR patients’ functional activities do not allow a quality of life, which is acceptable and/or pharmaceutical therapies have resulted in intolerable adverse effects.
- Psychiatric disorders and psychosocial factors that could influence pain outcomes have been assessed and appropriately addressed ^23, 24^; in practice, when the patient with cancer pain responds positively to any of these two questions:
  1. Is your pain under the current drug medication intolerable for your quality of life?
  2. Do you have intolerable adverse effects with your current treatment?
- Cancer pain classified as chronic (persistent or recurrent pain lasting longer than 3 months) ^25^, and currently refractory despite optimized analgesic therapy including an opioid. [Optimized analgesic therapy is arbitrarily defined as: oral morphine equivalent of 60 mg/d or more ^11, 26^ (or another strong opioid at optimized dose) plus at least one adjuvant analgesic drug, for at least 2 weeks.]
- No increase in baseline long acting opioid dose or addition of a new adjuvant analgesic drug within 2 weeks prior to study entry
- Ability to communicate the intensity of pain using the NPRS pain scale ranging from (0 as no pain to 10 with severe pain).
- Ability to give fully informed written consent.
- Expect survival more than 3 months.

*Exclusion criteria*, taking into consideration the safety profile of the drug ^22^ were defined as:

- History of allergy or intolerance to esketamine or ketamine.
- History of allergy to disinfecting products containing quaternary ammonium, who might be susceptible to be allergic to denatonium benzoate.
- Concomitant use of xanthine derivatives (e.g. aminophylline, theophylline), ergometrine, or monoamine oxidase inhibitors.
- Active nasal/sinus dysfunction (e.g. allergic or infectious rhinitis) or presence of any lesion of the nasal mucosa.
- Pregnancy, breastfeeding and women of childbearing potential not using a highly effective contraception method.
- Uncontrolled hypertension, arrhythmia, heart failure, or untreated coronary artery disease. History of transient ischemic attacks, stroke, neurovascular disease, hemorrhage, severe head injury, hydrocephalus or elevated intracranial pressure within the last 3 months.
- History of primary or metastatic malignant brain lesions (uncontrolled or without previous treatment).
- Known aneurysmal vascular disease (including thoracic and abdominal aorta, intracranial, and peripheral arterial vessels) or arteriovenous malformation
- Uncontrolled psychiatric illness with psychosis/hallucination (e.g. schizophrenia, acute psychosis).
- Alcohol abuse, drug abuse/dependence within the past 6 months as self-reported.
- Cirrhosis or severe hepatic impairment defined as 5-fold elevation of transaminases.
- Uncontrolled hyperthyroidism.
- Globe injuries or increased intraocular pressure (e.g. glaucoma).
- History of ulcerative or interstitial cystitis.
- Subjects scheduled to receive radiotherapy (RT) to a site of pain during the study period, or who have received RT to a site of pain within 2 weeks before study entry.
- Subjects scheduled to undergo surgical treatment during the study period likely to affect pain.
- Subjects on or starting chemotherapy if there is a significant expectation of that therapy affecting pain.
- Subjects who have not provided signed informed consent form.
- Concomitant use of drugs moderately or severely affecting cytochrome P450 activity.

### Recruitment strategy

Participants will be recruited at the oncology and pain clinics of a tertiary academic hospital by the attending physicians during routine appointments. Participants may also be identified via review of medical records from the oncology and pain clinics and contacted by telephone prior to the next scheduled appointment with information about the study, in order to allow more time for consideration. Screening will continue until the target sample size is achieved (120 subjects as calculated sample size, see below). Expected recruitment time will be two years.

### Adherence

To achieve adherence in the present clinical trial, the characteristics and main objective of the study will be exposed to the patients and their relatives who are in charge of their care during recruitment interviews and informed consent. An instructional video will be provided, which contains information about the correct drug administration, as well as the benefits of having a quick and easy application therapy. Adjuvant drugs currently used will be provided at each visit.

In the initial interview with the subject the following information will be given:

- Objectives of the study
- Mode of administration of interventions
- Importance of continuing with regular treatment (opioids, antidepressants, etc.)

### Timeline

**Figure 1.**
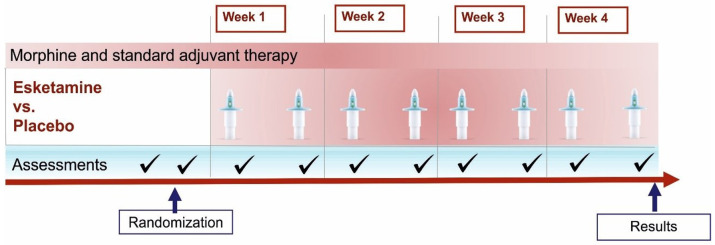
Timeline of scheduled visits and assessments. This figure displays the scheduled timeline of the study. Included are randomization, application of study drug/placebo (nasal spray devices), clinical assessments and time point of data collection (results).

Figure 1 demonstrates a timeline with concise description of scheduled visits and assessments.

### Interventions

The patients will be using around the clock baseline analgesia with long-acting opioid, and morphine rescue dose for the management of breakthrough pain. In the case of neuropathic pain, patients should start the use of an antidepressant or anticonvulsant in an optimized dose. If the participant is already using any of these drugs, they should continue the medication ^1^ .

The study drug will be provided in two disposable nasal spray devices containing either 28 mg of esketamine or placebo in a 200 uL of solution - equivalent to two sprays doses. The 28 mg esketamine nasal spray device labeled “Spravato ^TM^” and a similar placebo device should be provided by the manufacturer Janssen Pharmaceutical Company of Johnson & Johnson. The participants should initially self-administer the nasal spray device containing either active drug or placebo under the supervision of a health care provider in the researcher center. Health care providers (physicians or nurses) will be informed and trained prior to the beginning of recruitment. Training will be provided by the investigators. One spray must be administered into each nostril in a 45-degree reclination to deliver 28mg. After a 5 minute interval the second device is used to deliver the second 28mg dose or placebo ^22^.

The nasal spray device contains an indicator with two green dots referring to the two spray doses. After one spray is delivered, one green dot will disappear. When the device empties after the two doses, all green dots will have disappeared. The participants will receive the intervention twice weekly for 4 consecutive weeks.

### Outcomes

The primary outcome of this trial is a reduction in the 11-point Numeric Pain Rating Scale (NPRS). It will be used to assess pain intensity at enrollment and at each visit. Patients will be asked to rate their weekly pain on a scale from 0 to 10 where 0 equals “no pain” and 10 equals "the worst pain they can imagine” ^27^. NPRS will be taken for both during physical activity and at rest. The difference in NRPS after first dose of esketamine and baseline will be measured to analyze efficacy.

The use of a unidimensional measure is justified as the goal of this study is to evaluate the efficacy of the investigational drug as an analgesic complement for patients already consuming morphine. There are several tools to approach pain intensity and this choice (NRPS) was based on literature review on similar situations ^27– 30^.

Oncological patients suffering from chronic pain frequently present associated degrees of depression, which is why we chose a scale evaluating exclusively pain over other multidimensional questionnaires. This strategy enables to differentiate the analgesic action of esketamine from its role as antidepressant, as this itself might improve the patients’ overall well-being and disposition.

***Secondary outcomes.*** *Sustainability of effect.* NRPS will be used to identify the pattern of effect after each dose over the 4 weeks.

*Rescue morphine use*. The use of morphine rescue (whether it will be reduced, no change, or increased). If pain decreases or stabilizes during the trial. This will be monitored using either the Aircure artificial intelligence through a mobile application or personal diaries.

*Patients’ functional status and satisfaction.* These will be measured by the change in Brief Pain Inventory (BPI). A multidimensional standardized tool as the BPI will allow the researchers to assess pain intensity in relation to its interference in functional activities ^31^.

*Depression symptoms.* Depression is frequently described in association with chronic pain and affects its threshold. On the other hand, esketamine has a proven antidepressive effect. We propose to examine a depression score using the Patient Health Questionnaire (PHQ9) at enrollment and at each visit ^32^. We would also evaluate the effect of depression on the analgesic effect of esketamine.

*Side effects.* Reported side effects in other studies include decline in mood, conscious perceptions and intellectual performance. It is well established that esketamine presents fewer side effects than the racemic ketamine ^33^. In this protocol the dissociative effects of the drug will be monitored applying the Side Effect Rating Scale for Dissociative Anesthetics (SERSDA) at enrollment and at each visit ^34^. It assesses fatigue, dizziness, headache, nausea, changes in vision and mood changes. The cognitive performance will be addressed using Speed Reading Test (where the time for reading 20 independent characters is noted) ^33^.

*Vital signs.* Blood pressure, heart rate, respiratory rate and temperature, pulse oximetry and 12-lead-ECGs will be measured at enrollment and at each visit (prior, 40 minutes and 2 hours after intranasal application of the drug or placebo). In the occurrence of any abnormality, patients will be advised to stay at the institution for complementary evaluation. Furthermore, lower urinary tract symptoms will be asked and in case of positive answers, further examination will be performed ^35^.

### Data monitoring

A DMC will be established, which is independent of the trial sponsor and study investigators. The DMC will be composed by experts of the field related to the study ^36^ (at least 1 oncologist, 1 anesthesiologist, 1 psychiatrist, 1 pharmacologist, 1 patient advocacy, and 1 statistician). All members of the DMC will have to declare competing interests. Besides, the DMC will also monitor recruitment, inclusion rate, baseline characteristics, participant adherence, adverse events and safety monitoring every 15 days confidentially ^37^.

### Harms

Solicited and spontaneously reported adverse events will be collected by the outcome assessors at each planned visit for a clinical assessment. Details about efficacy and safety of the trial medication have been reported previously (see above).

### Sample size calculation

Sample size is based on practical and clinical considerations for this phase II trial. Based on a previous study (case series) assessing the analgesic effect of nasal esketamine ^38^ we obtained a standard deviation of 5.5 (that is the 75% upper limit of the confidence interval of the standard deviation). However, we would like to emphasize that power alone will not be relevant for the clinical interpretation of the study results, especially in a clinical trial ^39, 40^.

Considering the population of patients with refractory cancer pain (20%), to achieve a primary outcome reduction of 3 points on the 11 points NPRS with a power of 80% and an alpha error of 5%, a sample size of 108 subjects is needed. Based on previous studies in this patient population a dropout rate of 10% is estimated ^39, 41^, so for reasons of compensation, a total sample size of 120 participants will be recruited ^42, 43^.

### Statistical analysis plan

For the purpose of descriptive statistics all numerical continuous data will be presented as means or medians with standard deviation (SD) and interquartile ranges (IQR) respectively, as measures of dispersion. Categorical data will be presented in the form of proportions, frequencies or percentages. For all primary and secondary analysis, the intervention arm (nasal esketamine plus opioid therapy plus adjuvant standard analgesia) will be compared with the control group (placebo plus opioid therapy plus adjuvant standard therapy).

The P-values will be reported with two decimal points; all our tests will be 2-sided p-values with a level of significance of <0.05 to consider statistical significance. The statistical software to be employed is an up-to-date version of STATA (StataCorp LLC, College Station, Texas, USA), managed by professional blinded statisticians. After the analysis of all data, if the effect of esketamine doesn't last until next dose, a clustered analysis might be performed using results from all visits to evaluate acute efficacy of the drug against placebo.

***Missing data.*** According to our sample size calculation, we are considering an estimation of missing data of 10% of the participants, which may leave the study for several reasons that can include death secondary to cancer, adverse reactions, lack of improvement, early recovery, and others ^44, 45^.

## Ethical considerations

### Research ethics approval

Research ethics committee/institutional review board (REC/IRB) approval will be obtained by the local ethics committee or institutional review board of the site center prior to begin of recruitment.

### Protocol amendments

Protocol amendments, if any, should be registered and approved by the local ethics committee. Accordingly, study protocol and if applicable informed consent form will be adjusted and investigators involved in the trial informed immediately.

## Dissemination

After completion of data acquisition and analysis, the results will be summarized and published. Accordingly, patients will be informed about allocation and trial results via telephone calls and optional visit in person. Manuscript writing will be performed by the investigators and there will be no public access granted to the raw data.

### Confidentiality

Personal information about enrolled patients will be collected by study nurses of the study site. Collected information will be saved in coded folders and investigators, outcome assessors and statisticians will receive a pseudononymized version of the data. The data and information will be stored at a previously defined location after completion of the trial for 5 additional years.

### Access to data

Only investigators and statisticians will have access to the final version of the data. To secure restricted access to the data, corresponding files will be coded.

### Ancillary and post-trial care

Meanwhile and after completion of the trial patients will be treated according to current guidelines and standards of care. Any harm associated with the participation of the trial is not expected as patients continue to receive standard of care.

## Study status

Not yet recruiting.

## Data availability

### Underlying data

No data is associated with this article.

### Reporting guidelines

Figshare: SPIRIT and TIDieR checklist for ‘IMPORTANCE trial: a provisional study-design of a single-center, phase II, double-blinded, placebo-controlled, randomized, 4-week study to compare the efficacy and safety of intranasal esketamine in chronic opioid refractory pain’, https://doi.org/10.6084/m9.figshare.13435742.v2 ^46^.

Data are available under the terms of the Creative Commons Zero "No rights reserved" data waiver (CC0 1.0 Public domain dedication).
