# Supplementary material for: IMPORTANCE trial: a provisional study-design of a single-center, phase II, double-blinded, placebo-controlled, randomized, 4-week study to compare the efficacy and safety of intranasal esketamine in chronic opioid refractory pain
Source: F1000Res. 2021 Jan 22;10:42. [Version 1] doi: 10.12688/f1000research.27809.1 (PMC7885290; doi:10.12688/f1000research.27809.1)
Supplement: Peer review history

# Peer review: Reviewer response for version 1

**DOI:** 10.5256/f1000research.30754.r79031

**Published:** 2021-02-15

**Authors:** Danny Kupka

**Affiliations:** 1 Medical Oncology and Hematology, University Hospital Zurich, Zurich, Switzerland

The article represents the protocol of the I **MPORTANCE trial**. The IMPORTANCE trial (phase II, randomized and double-blind)  evaluates the efficacy and safety of intranasal administration of S-Ketamine in chronic opioid refractory cancer pain. The primary outcome is the reduction in the Numeric Pain Rating Scale. 

 The study is well designed with appropriate questionnaires to determine primary and secondary outcomes. 

 For me, sample size calculation based on ref. 38 is possible but addresses a different patient set. Maybe the authors comment on that briefly. 

 Moreover, I would comment on the fact that no midazolam is used in combination with S-Ketamin. However, this would imply 3 study groups (S-Ketamine + Midazolam i.n. versus Midazolam i.n. versus placebo). 

 The manuscript sounds scientific. I have no additional comments on this issue.

Is the study design appropriate for the research question?

Yes

Is the rationale for, and objectives of, the study clearly described?

Yes

Are sufficient details of the methods provided to allow replication by others?

Yes

Are the datasets clearly presented in a useable and accessible format?

Not applicable

Reviewer Expertise:

Cardiology, Oncology, Immunology

I confirm that I have read this submission and believe that I have an appropriate level of expertise to confirm that it is of an acceptable scientific standard.

# Peer review: Reviewer response for version 1

**DOI:** 10.5256/f1000research.30754.r77932

**Published:** 2021-02-05

**Authors:** Vinita Singh

**Affiliations:** 1 Department of Anesthesiology, Emory University School of Medicine, Atlanta, GA, USA

This is a well-written description of a prospective clinical trial that will test the efficacy of intranasal esketamine in the reduction of chronic, refractory, cancer pain. A total of 120 patients will be recruited and randomized to receive placebo or intranasal esketamine 56 mg twice a week for 4 weeks. The primary outcome is a reduction in pain score, after the first dose of medication, on Numerical Pain Rating Scale. There is an appropriate data safety monitoring plan.

 Few critiques below:

1. PHQ-9 is a screening tool for depression, but not measuring changes in depression.
2. There is a section for missing data, however, how missing data will be handled is not specified. Also, there's can be several reasons for missing data besides patients leaving the study.

Is the study design appropriate for the research question?

Yes

Is the rationale for, and objectives of, the study clearly described?

Yes

Are sufficient details of the methods provided to allow replication by others?

Yes

Are the datasets clearly presented in a useable and accessible format?

Yes

Reviewer Expertise:

Intranasal ketamine for cancer and chronic neuropathic pain, cancer pain, neuropathic pain, non-opioid and non-pharmacological treatment for cancer pain

I confirm that I have read this submission and believe that I have an appropriate level of expertise to confirm that it is of an acceptable scientific standard.
